# Clinical utility of the PBAC score in quantifying treatment response to hysteroscopy: a retrospective observational study

**DOI:** 10.3389/fsurg.2025.1684443

**Published:** 2025-11-28

**Authors:** Ömer Faruk Öz, Can Dinç, Burhan Öğüt, Saltuk Buğra Arıkan, Selen Doğan, Murat Özekinci, İnanç Mendilcioğlu

**Affiliations:** 1 Department of Obstetrics and Gynecology, Akdeniz University Faculty of Medicine, Antalya, Türkiye; 2 Department of Obstetrics and Gynecology, Akdeniz University Hospital, Antalya, Türkiye

**Keywords:** pictorial blood loss assessment chart (PBAC), hysteroscopy, abnormal uterine bleeding, minimally invasive surgery, patient satisfaction

## Abstract

**Objective:**

To evaluate the clinical utility of the Pictorial Blood Loss Assessment Chart (PBAC) as a quantitative tool for measuring treatment response in patients undergoing hysteroscopy for abnormal uterine bleeding (AUB), and to examine its correlation with changes in hemoglobin levels, endometrial thickness, and patient satisfaction.

**Materials and methods:**

This retrospective study included 327 patients who underwent hysteroscopy for AUB between January 2021 and January 2024 at a tertiary care center. Patients with incomplete records, inadequate follow-up, technically unsuccessful procedures, concurrent major surgeries, or endometrial cancer were excluded. Postoperative menstrual bleeding was assessed using the Pictorial Blood Loss Assessment Chart (PBAC). Data were analyzed using SPSS version 26, and *p* < 0.05 was considered statistically significant.

**Results:**

Of the 327 patients included in the study, 91.1% had a baseline PBAC score ≥100, which significantly decreased following hysteroscopy (mean reduction: 393 points; *p* < .001). Postoperative Hemoglobin (Hb) levels and Endometrial Thickness (ET) also improved significantly in patients with high initial bleeding scores. Overall complication rate was low (6.4%), and 87% of patients reported satisfaction and willingness to recommend the procedure. McNemar analysis confirmed a significant shift from high to low bleeding severity postoperatively (*p* < .001).

**Conclusions:**

Hysteroscopy demonstrated clinical efficacy in the management of abnormal uterine bleeding. Assessment using the PBAC score provided a feasible and informative method for quantifying treatment response, supporting its role as a valuable tool in the objective evaluation of bleeding outcomes.

## Introduction

Abnormal uterine bleeding (AUB) is a common gynecological condition encountered in clinical practice, affecting women across various age groups and significantly impairing quality of life, daily functioning, and sexual well-being (1, 2). The differential diagnosis of AUB includes a wide range of structural and non-structural etiologies, as outlined in the PALM-COEIN classification system (1). Establishing an accurate diagnosis and providing effective management requires an interdisciplinary approach involving clinical evaluation, imaging techniques, and, in selected cases, direct visualization of the uterine cavity.

Hysteroscopy has become the gold-standard diagnostic and therapeutic tool for evaluating intrauterine pathologies, particularly in women with abnormal uterine or postmenopausal bleeding (PMB) (2). In addition to enabling direct visualization, it allows precise therapeutic procedures such as polypectomy, myomectomy, and endometrial ablation, all performed in a minimally invasive manner. Studies conducted in both outpatient and office-based settings have confirmed the safety, effectiveness, and high satisfaction rates associated with operative hysteroscopy (3).

While the diagnostic accuracy of hysteroscopy is well-established, limited evidence exists regarding its actual impact on menstrual symptomatology, particularly when evaluated using objective measurement tools (4). One such tool is the Pictorial Blood Loss Assessment Chart (PBAC), a validated semi-quantitative method commonly employed in research settings to assess the severity of menstrual bleeding in women with AUB (5). Originally developed by Higham et al. (6), the PBAC assigns scores based on the degree of soiling on sanitary products, with a threshold of ≥100 correlating with objectively measured menstrual blood loss exceeding 80 mL, which is a widely accepted criterion for menorrhagia. The tool has demonstrated high sensitivity and specificity in identifying heavy menstrual bleeding (6), yet it remains underutilized in routine assessments of hysteroscopic outcomes.

Subsequent studies have confirmed the diagnostic validity of PBAC in various populations. For instance, Ko et al. reported excellent discriminatory capacity between normal and excessive menstrual bleeding among Asian women, with an area under the curve (AUC) of 0.96, reinforcing its reliability as a screening instrument in clinical practice (7). Moreover, PBAC has also proven useful for monitoring treatment outcomes in AUB. A reduction of more than 100 points in PBAC scores following intervention has been associated with clinically meaningful improvements in Hb levels and patient-reported quality of life (7).

In this study, we aimed to explore how hysteroscopy impacts clinical outcomes in women undergoing the procedure for abnormal uterine or postmenopausal bleeding. We hypothesized that changes in PBAC scores following hysteroscopy would be associated with improvements in hemoglobin levels, endometrial thickness, and patient-reported satisfaction.

## Materials and methods

This retrospective observational study was conducted at Akdeniz University, Department of Obstetrics and Gynecology, with ethics approval from the Ethics Committee of Akdeniz University Tıbbi Bilimsel Araştırmalar Etik Kurulu (Akdeniz University Medical Scientific Research Ethics Committee), approval number: TBAEK-243. All procedures were conducted in accordance with relevant ethical guidelines and regulations, including the Declaration of Helsinki. Informed consent was obtained from all participants prior to inclusion in the study. The study was reported in line with the STROBE guidelines.

Patients undergoing hysteroscopy for AUB or PMB between January 2021 and December 2024 were screened (*n* = 419); 327 met eligibility criteria after exclusions. To minimize potential sources of bias, standardized data collection was applied across all patients, and those with incomplete data or inadequate follow-up were excluded. No formal sample size calculation was performed. All eligible patients who met the inclusion criteria during the study period were included to enhance the representativeness of the data. As a result, no missing data were present for the analyzed variables.

Exclusion criteria included incomplete data, concurrent surgeries, hormonal therapy, bleeding disorders, pregnancy-related bleeding, malignancy, or major uterine anomalies. Data were retrieved from electronic records and supplemented via follow-up at 3 months through in-person visits or phone calls. Participants completed pre- and postoperative PBAC forms using the standardized Higham PBAC chart (6). A facsimile of the chart with detailed scoring instructions is provided in Supplementary Figure S1. PBAC scores were grouped using a cutoff of 100, the validated threshold for menorrhagia (6, 8).

PBAC scoring was performed using the standardized Higham chart (Supplementary Figure S1, adapted from Higham et al., 1990). At enrollment, patients received verbal instruction, an illustrated leaflet, and a sample filled form to ensure correct use. A trained nurse verified understanding before study entry. The reliability and validity of the PBAC system in assessing AUB have been demonstrated in previous studies (Higham et al., 1990; Janssen et al., 1995). For quality control, patients recorded bleeding data over three consecutive menstrual cycles, starting from the last complete preoperative cycle and including the first two complete postoperative cycles. Bleeding was categorized according to spotting, normal flow, and heavy flow (clots/flooding) in line with PBAC definitions. Diaries were reviewed at follow-up visits, and incomplete or inconsistent entries were reconciled through patient interviews or phone verification. Missing days were coded as zero if absence of bleeding was confirmed; otherwise, they were excluded from analysis.

Outcomes and definitions. Complications within 30 days were classified as endometritis (fever, pelvic pain, abnormal discharge plus positive culture), uterine perforation (intraoperative or radiological confirmation), or significant postoperative bleeding (requiring intervention). Patient-reported outcomes were dichotomized as satisfaction (Likert 0–3; satisfied = 2–3) and willingness to recommend (yes/no). Cervical-ripening agent use, subsequent surgery, and histopathology were recorded.

Statistical analyses were performed in SPSS v26 (IBM Corp., Armonk, NY, USA). Normality was assessed visually (histograms, Q–Q plots) and analytically (Shapiro–Wilk). Paired baseline-to-postoperative comparisons for PBAC, hemoglobin (Hb), and endometrial thickness (ET) used the Wilcoxon signed-rank test (two-sided). Between-group comparisons used the Mann–Whitney *U* test for continuous data and *χ*² or Fisher's exact tests for categorical data; McNemar's test was used for paired binary outcomes when applicable. Correlations between continuous variables were assessed with Spearman's *ρ* (primary), with Pearson's r examined as sensitivity; scatter plots with least-squares trend lines are provided for visualization.

For consistency, ΔPBAC was defined as postoperative minus baseline (negative values indicate improvement); when “PBAC reduction” is reported, it refers to—ΔPBAC. For binary outcomes (complications, satisfaction, recommendation), unadjusted odds ratios (ORs) with 95% confidence intervals were calculated from 2 × 2 tables and displayed in a forest plot (Supplementary Figure S2). Statistical significance was set at two-sided *p* < 0.05. Given the retrospective, primarily descriptive design, no multivariable modeling was performed.

**Figure 1:**
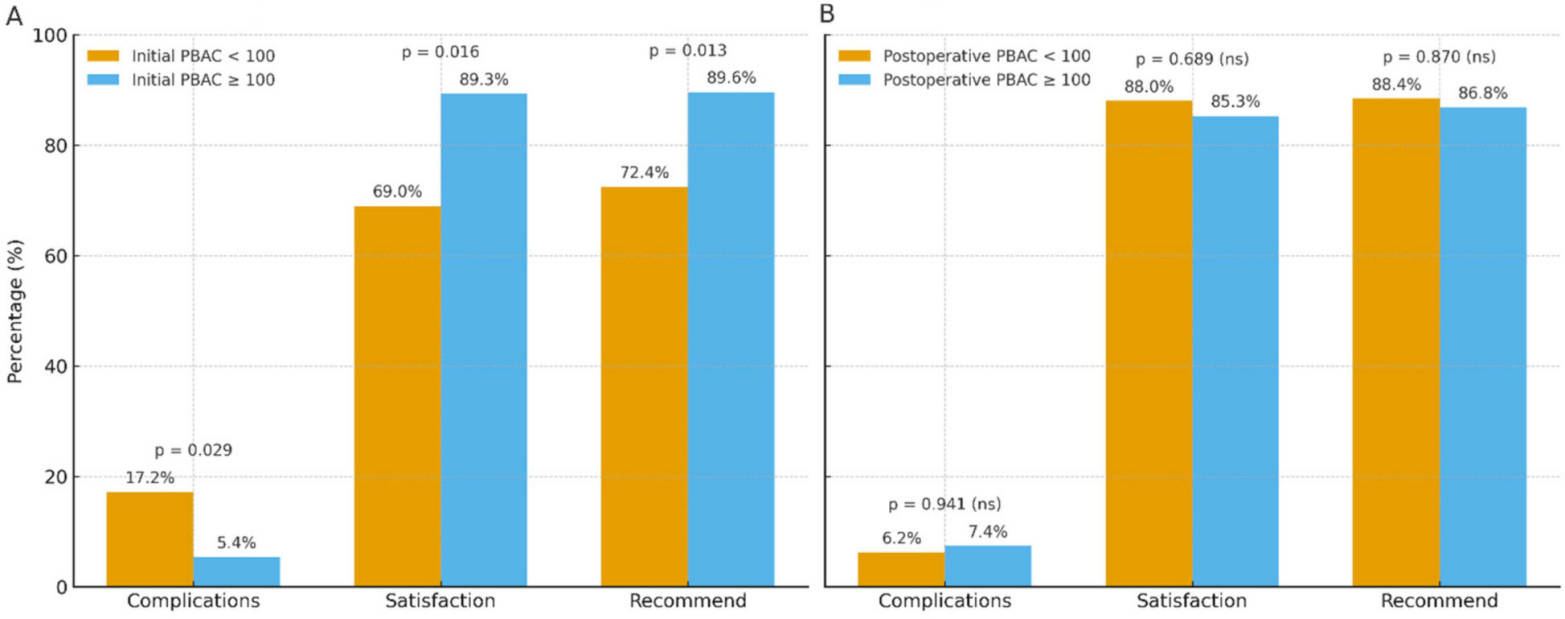
Patient outcomes by PBAC grouping. **(A)** Outcomes by initial PBAC (<100 vs. ≥100): complications, satisfaction, and willingness to recommend (*p* = 0.029, 0.016, 0.013, respectively). **(B)** Outcomes by postoperative PBAC (<100 vs. ≥100): no significant between-group differences (all *p* > 0.05). Bars show percentages with value labels; *p*-values are from two-sided *χ*² or Fisher's exact tests. Counts and exact percentages are provided in Table 3.

Presentation of categorical data. Procedural and histopathology variables are summarized as counts (%). To avoid double-counting when multiple findings co-occurred, a prespecified hierarchy assigned a single primary category: procedures—myomectomy > endomyometrial resection > polypectomy; histopathology—EIN/atypical hyperplasia > non-atypical hyperplasia > leiomyoma > endometrial polyp > adenomyosis > endometritis > normal > other. Group comparisons used *χ*² or Fisher's exact tests, as appropriate. Exact percentages with *p*-values are displayed in Figure 1; non-significant results are labeled “ns”.

Data availability. The datasets analyzed during the current study are available from the corresponding author on reasonable request.

## Results

The study included 327 patients with a mean age of 43.5 years and BMI of 27.6 kg/m², indicating an overweight profile. Most hospital stays were short (median: 8 h), although a small proportion of patients had extended admissions, resulting in a right-skewed distribution.

Mean baseline hemoglobin (Hb) was 10.2 g/dL, increasing to 11.0 g/dL postoperatively. Endometrial thickness (ET) decreased from 13.6 mm to 7.1 mm following treatment. The average surgery duration was 23.3 min, and mean fluid deficit was 634.8 cc, with a broad range (150–1,850 cc).

**Figure 2:**
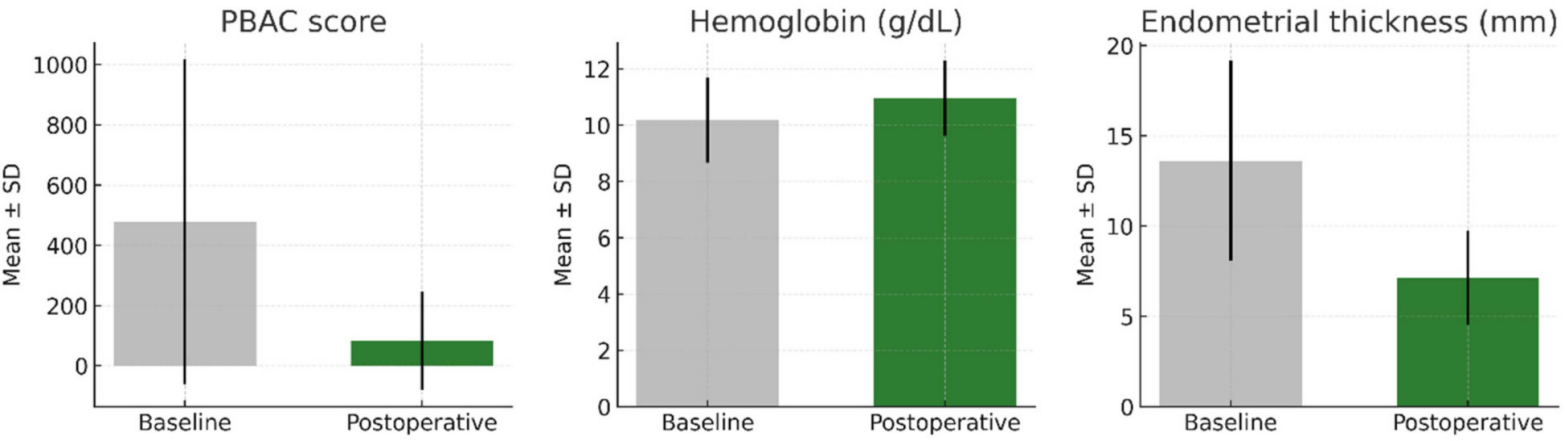
Baseline–postoperative changes in PBAC score, hemoglobin, and endometrial thickness. Grouped bar charts show mean ± SD at baseline and postoperatively. All paired comparisons were statistically significant (*p* < 0.001).

PBAC scores showed a mean reduction of 393 points (from 477 to 84), with high baseline variability suggesting a subgroup of patients experienced severe menorrhagia preoperatively (see Table 1). From baseline to the first postoperative assessment, the PBAC score and endometrial thickness declined substantially, whereas hemoglobin increased. The direction and magnitude of change are visualized in Figure 2 (grouped bar charts showing mean ± SD), and all paired comparisons were statistically significant (all *p* < 0.001).

**Table 1:** Descriptive statistics for continuous variables.

*n* = 327	Mean ± SD	Median (Q1-Q3)	95% CI
Age	43.5 ± 7.5	43 (39–48)	42.69–44.31
Length of Hospital Stay (hours)	20.5 ± 27.1	8 (8–24)	17.56–23.44
BMI, kg/m²	27.6 ± 4.2	27 (24.7–30.3)	27.14–28.06
Baseline Hb (g/dL)	10.2 ± 1.5	10.2 (9.2–11.2)	10.04–10.36
Follow-up Hb (g/dL)	10.9 ± 1.4	10.8 (10.3–12.2)	10.75–11.05
Initial PBAC Score	477.2 ± 539.4	330 (186–475)	418.74–535.66
Follow-up PBAC Score	83.7 ± 163.9	45 (25–90)	65.94–101.46
Preoperative ET (mm)	13.6 ± 5.6	13 (9–18)	12.99–14.21
Postoperative ET (mm)	7.2 ± 2.62	7 (5–8)	6.92–7.48
Duration of Surgery (minutes)	23.3 ± 5.3	24 (19–27)	22.73–23.87
Fluid Deficit (cc)	634.8 ± 328.01	550 (400–780)	599.25–670.35
Change in PBAC Score	393.5 ± 541.6	230 (130–400)	334.80–452.20
Change in Hb (g/dL)	0.8 ± 1.01	0,9 (0.3–1.3)	0.69–0.91
Change in ET (mm)	−6.5 ± 5.5	−6 (−10–−3)	−7.10–−5.90

This was further supported by detailed comparisons including 95% confidence intervals. The mean PBAC score significantly decreased from 477.2 (95% CI: 418.7–535.7) to 83.7 (95% CI: 65.9–101.5), *p* < 0.001. Mean hemoglobin level increased from 10.2 g/dL (95% CI: 10.1–10.3) to 10.9 g/dL (95% CI: 10.8–11.0), *p* < 0.001. Endometrial thickness also showed a significant reduction from 13.6 mm (95% CI: 13.1–14.1) to 7.2 mm (95% CI: 6.9–7.5), *p* < 0.001.

These findings are detailed in Table 1. Statistically significant differences were observed between patients with baseline PBAC scores <100 and ≥100. Age, hospital stay, follow-up hemoglobin, surgery duration, fluid deficit, and change in endometrial thickness did not differ significantly between groups (*p* > .05).

Patients with PBAC < 100 had significantly higher BMI (*p* = .022) and baseline hemoglobin (*p* = .033). Although their follow-up PBAC scores were lower (*p* < .001), the ≥100 group exhibited greater PBAC reductions (*p* < .001) and hemoglobin increases (*p* = .034), reflecting more pronounced treatment response in those with heavier initial bleeding.

Table 2 compares patients based on follow-up PBAC scores (<100 vs. ≥100). Those with follow-up PBAC ≥ 100 had significantly higher baseline scores (M = 628.8 vs. 437.4, *p* < .001) and follow-up scores (M = 255.7 vs. 38.5, *p* < .001). No other significant differences were observed in demographic or perioperative variables (*p* > .05).

**Table 2:** Comparison of clinical parameters according to initial and follow-up PBAC.

Clinical parameter	Initial PBAC < 100 (*n* = 29)	Initial PBAC ≥ 100 (*n* = 298)	Follow-up PBAC < 100 (*n* = 259)	Follow-up PBAC ≥ 100 (*n* = 68)	*p* (Initial PBAC groups)	*p* (Follow-up PBAC groups)
Age	45.5 ± 7.5/46 (41–49)	43.3 ± 7.5/43 (39–48)	43.9 ± 7.7/43 (39–48)	42.1 ± 6.6/42 (39–47)	0.122	0.219
Length of Hospital Stay (hours)	19.9 ± 21.3/8 (8–24)	20.5 ± 27.6/8 (8–24)	20.3 ± 26.1/8 (8–24)	21.1 ± 30.6/8 (8–24)	0.737	0.682
BMI (kg/m^2^)	29.8 ± 5.8/30 (24.9–34.4)	27.4 ± 3.9/27 (24.6–30.2)	27.6 ± 4.2/27 (24.7–30.2)	27.3 ± 4.0/27.2 (24.7–30.8)	**0** **.** **022**	0.582
Baseline Hb (g/dL)	11.0 ± 2/10.4 (9.5–13.2)	10.1 ± 1.4/10.2 (9.2–11.1)	10.2 ± 1.5/10.2 (9.2–11.2)	10.2 ± 1.5/10.2 (9.4–11.2)	**0** **.** **033**	0.824
Follow-up Hb (g/dL)	11.4 ± 1.8/11.4 (10–12.7)	10.9 ± 1.3/10.8 (10.4–11.8)	11.0 ± 1.4/10.8 (10.2–12.3)	10.9 ± 1.2/10.9 (10.4–11.7)	0.186	0.938
Initial PBAC Score	58.2 ± 21.9/60 (44–75)	517.9 ± 548.1/340 (230–540)	437.4 ± 525.7/270 (165–440)	628.8 ± 567.5/414.5 (305–755)	**<0** **.** **001**	**<0** **.** **001**
Follow-up PBAC Score	32.8 ± 25.6/30 (15–43)	88.6 ± 170.7/45 (25–90)	38.5 ± 23.2/35 (20–50)	255.7 ± 301.1/150 (130–300)	**<0** **.** **001**	**<0** **.** **001**
Preoperative ET (mm)	12.6 ± 5.5/12 (8–16)	13.7 ± 5.6/13.2 (9–18)	13.6 ± 5.6/13 (9–18)	13.5 ± 5.5/13.5 (9–17)	0.338	0.988
Postoperative ET (mm)	6.3 ± 2.4/6 (5–8)	7.2 ± 2.6/7 (5–8)	7.0 ± 2.4/7 (5–8)	7.5 ± 3.3/7 (5–8.5)	0.074	0.629
Duration of Surgery (minutes)	21.9 ± 4.6/22 (19–25)	23.5 ± 5.3/24 (19–27)	23.2 ± 5.3/24 (19–27)	23.6 ± 5.0/24 (19.5–27)	0.106	0.716
Fluid Deficit (cc)	568.3 ± 266.5/500 (400–600)	641.3 ± 333.1/550 (400–800)	626.8 ± 326.7/550 (400–750)	665.4 ± 333.6/600 (450–850)	0.329	0.259
Change in PBAC Score	25.5 ± 23.5/27 (5–36)	429.3 ± 554.4/274.5 (150–424)	398.9 ± 524.8/241 (134–395)	373.1 ± 604.8/200 (105–415)	**<0** **.** **001**	0.323
Change in Hb (g/dL)	0.4 ± 1.1/0.5 (–0.6–1.2)	0.8 ± 1.0/0.9 (0.3–1.3)	0.8 ± 1.0/0.9 (0.3–1.3)	0.7 ± 1.0/1 (0.2–1.3)	**0** **.** **034**	0.89
Change in ET (mm)	−6.26 ± 5.6/−6 (–10––1.0)	−6.51 ± 5.5/−6 (–10––3)	−6.6 ± 5.2/−6 (–10––3)	−6.1 ± 6.3/−6 (–10––2)	0.792	0.605

Bold values indicate statistically significant differences (*p* < 0.05).

Overall, 91.1% of patients initially had PBAC ≥ 100, decreasing to 20.8% at follow-up, indicating substantial symptom improvement. Cervical ripening agents were used in 51.1% of cases. The most common procedure was endomyometrial resection (71.9%), followed by polypectomy (19.0%) and myomectomy (12.5%).

Histopathologic findings included normal endometrium (36.7%), endometrial polyps (21.4%), and leiomyomas (15.0%). Less common diagnoses were adenomyosis, hyperplasias, EIN, and endometritis.

**Figure 3:**
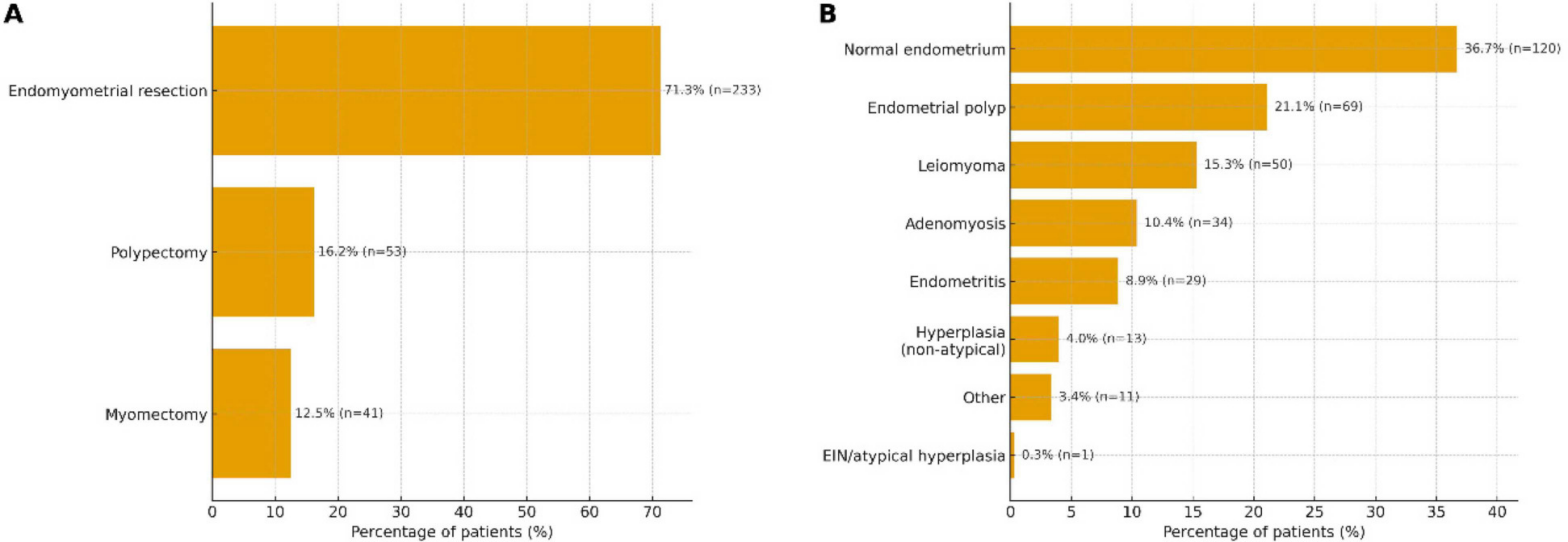
Distribution of surgical procedures **(A)** and histopathological findings **(B)** (*n* = 327). Horizontal bars display percentages with counts for each category. To avoid double-counting, a single primary assignment per patient was used based on a prespecified hierarchy (procedures: myomectomy > endomyometrial resection > polypectomy; histopathology: EIN/atypical hyperplasia > non-atypical hyperplasia > leiomyoma > endometrial polyp > adenomyosis > endometritis > normal > other). These displays summarize the overall case-mix within the cohort.

Figure 3 depicts the distribution of surgical procedures and histopathological findings in the cohort. Endomyometrial resection constituted the largest share of procedures, followed by polypectomy and myomectomy; benign histopathology predominated, with normal endometrium and endometrial polyp among the most frequent categories. Bars indicate percentages with counts (n), using a single primary assignment per patient to avoid double-counting (Figure 3).

The overall complication rate was low (6.4%), with bleeding (3.1%) being the most frequent, followed by perforation and endometritis. Additional surgery was required in 24.2% of cases, most commonly hysterectomy.

Grouping by initial PBAC showed higher complications and lower patient-reported outcomes in those with PBAC < 100 at baseline (17.2% vs. 5.4%, 69.0% vs. 89.3%, 72.4% vs. 89.6%; *p* = 0.029, 0.016, 0.013; Figure 1A; Table 3).

**Table 3:** Descriptive statistics and comparisons of categorical clinical variables by initial PBAC group and by postoperative PBAC group (≥100 vs. <100).

Clinical variable	Category	Initial PBAC <100 (*n* = 29)	Initial PBAC <100 (%)	Initial PBAC > = 100 (*n* = 298)	Initial PBAC > = 100 (%)	Follow-up PBAC <100 (*n* = 259)	Follow-up PBAC <100 (%)	Follow-up PBAC > = 100 (*n* = 68)	Follow-up PBAC > = 100 (%)	*p* (Initial PBAC)	*p* (Follow-up PBAC)
Cervical Ripening	Not used	16	55.20%	144	48.30%	124	47.90%	36	52.90%	0.481^a^	0.457^a^
	Used	13	44.80%	154	51.70%	135	52.10%	32	47.10%		
Pathology	Normal Endometrium	11	37.90%	109	36.60%	94	36.30%	26	38.20%	0.385^b^	0.995^b^
	Endometrial Polyp	10	34.50%	60	20.10%	55	21.20%	15	22.10%		
	Leiomyoma	4	13.80%	45	15.10%	39	15.10%	10	14.70%		
	Adenomyosis	0	0.00%	34	11.40%	27	10.40%	7	10.30%		
	Complex Hyperplasia	1	3.40%	12	4.00%	11	4.20%	2	2.90%		
	Endometritis	0	0.00%	1	0.30%	1	0.40%	0	0.00%		
	Simple Hyperplasia	2	6.90%	27	9.10%	24	9.30%	5	7.40%		
	EIN	1	3.40%	10	3.40%	8	3.10%	3	4.40%		
Satisfaction (Three-Level)	Not at all satisfied-	9	31%^c^	32	10.7%	31	12%	10	14.7%	**0.016** **^a^**	0.832^a^
	Not satisfied										
	Satisfied	10	34.50%^c^	138	46.30%	118	45.60%	30	44.10%		
	Very satisfied	10	34.50%^c^	128	43.00%	110	42.50%	28	41.20%		
Willingness to Recommend	No	8	27.60%	31	10.40%	30	11.60%	9	13.20%	**0** **.** **013** **^b^**	0.708^a^
	Yes	21	72.40%	267	89.60%	229	88.40%	59	86.80%		
Procedure: EMR	Not performed	7	24.10%	85	28.50%	69	26.60%	23	33.80%	0.616^a^	0.241^a^
	Performed	22	75.90%	213	71.50%	190	73.40%	45	66.20%		
Procedure: Polypectomy	Not performed	24	82.80%	241	80.90%	214	82.60%	51	75.00%	0.805^a^	0.153^a^
	Performed	5	17.20%	57	19.10%	45	17.40%	17	25.00%		
Procedure: Myomectomy	Not performed	26	89.70%	260	87.20%	227	87.60%	59	86.80%	0.999^b^	0.845^a^
	Performed	3	10.30%	38	12.80%	32	12.40%	9	13.20%		
Overall Complication Rate	No	24	82.80%	282	94.60%	243	93.80%	63	92.60%	**0** **.** **029** **^b^**	0.781^b^
	Yes	5	17.20%	16	5.40%	16	6.20%	5	7.40%		
Postoperative Surgery	Not Performed	25	86.20%	223	74.80%	195	75.30%	53	77.90%	0.172^b^	0.850
	Performed	4	13.80%	75	25.20%	64	24.70%	15	22.10%		

Bold values indicate statistically significant differences (*p* < 0.05).

a Pearson's Chi-Square Test.

b Fisher's Exact Test.

c Indicates that values within the same column differ significantly from each other (*p* < 0.05; post hoc comparison).

In contrast, when grouped by postoperative PBAC, complications, satisfaction, and willingness to recommend did not differ between categories (all *p* > 0.05; Figure 1B; Table 3).

As shown in Figure 1A and Table 3, satisfaction levels differed significantly between initial PBAC groups (31.0% vs. 10.7% dissatisfaction; *p* = .016). Similarly, willingness to recommend was higher in the initial PBAC ≥ 100 group (89.6% vs. 72.4%; *p* = .013).

Complication rates also varied significantly (*p* = .029), occurring in 17.2% of the PBAC < 100 group compared to 5.4% of the ≥100 group. Notably, uterine perforation was more frequent in the <100 group (10.3% vs. 1.0%, *p* = .011). No other categorical variables showed significant group differences, including cervical ripening, pathology, type of surgery, postoperative reintervention, or endometrial findings (*p* > .05). These between-group differences are visualized in Figure 1A.

When stratified by follow-up PBAC scores (<100 vs. ≥100), no significant differences were observed in categorical clinical variables (Table 4, *p* > .05). Use of cervical ripening agents was comparable (52.1% vs. 47.1%, *p* = .457), and normal endometrium was the most common histopathological finding in both groups (36.3% vs. 38.2%, *p* = .995).

**Table 4:** Change in PBAC category (≥100 vs. <100) from baseline to postoperative (McNemar test).

Follow-up PBAC Score			Inıtial PBAC Score		Total	*p*
			<100	> = 100		
Follow-up PBAC Score	<100	*n*	29	230	259	**<0** **.** **001**
		% within Follow-up PBAC Score Group	11.2%	88.8%	100.0%	
		% within Initial PBAC Score Group	100.0%	77.2%	79.2%	
	> = 100	*n*	0	68	68	
		% within Follow-up PBAC Score Group	0.0%	100.0%	100.%	
		% within Initial PBAC Score Group	0.0%	22.8%	**20**.**8%**	
Total		Count	29	298	327	
		% within Follow-up PBAC Score Group	8.9%	**91**.**1%**	100.0%	
		% within Initial PBAC Score Group	100.0%	100.0%	100.0%	

Statistical analysis was performed using the McNemar test.

Bold values indicate statistically significant change in PBAC category from baseline to postoperative (*p* < 0.05; McNemar test).

Patient satisfaction and willingness to recommend the procedure were similarly high across groups. Dissatisfaction rates were low and statistically similar (12.0% vs. 14.7%, *p* = .832), as were recommendation rates (88.4% vs. 86.8%, *p* = .708).

Surgical techniques, including EME, polypectomy, and myomectomy, were performed at similar rates between PBAC groups. For example, polypectomy was applied in 17.4% vs. 25.0% of patients (*p* = .153). Complication rates were low and comparable (93.8% vs. 92.6% without complications, *p* = .691), and no significant differences were observed in complication types. Likewise, the need for further surgery did not differ between groups (24.7% vs. 22.1%, *p* = .850).

Among patients with initial PBAC ≥ 100, 77.2% improved to <100 at follow-up, whereas none in the <100 group worsened. This asymmetry was significant (McNemar *p* < .001), indicating treatment-related reduction in bleeding. Overall, PBAC ≥ 100 prevalence dropped from 91.1% at baseline to 20.8% at follow-up (McNemar *p* < .001; Table 4).

Table 5 summarizes pre- and post-treatment changes in hemoglobin and endometrial thickness (ET) based on initial PBAC scores.

**Table 5:** Comparison of hemoglobin and endometrial thickness (ET) changes according to PBAC score groups.

Clinical variable	Initial PBAC < 100 (*n* = 29)	Initial PBAC ≥ 100 (*n* = 298)	Follow-up PBAC <100 (*n* = 259)	Follow-up ≥ 100 (*n* = 68)
Hb (g/dL)	**Mean ± SD**	**Mean ± SD**	**Mean ± SD**	**Mean ± SD**
Basal	11.0 ± 2.0	10.1 ± 1.4	10.2 ± 1.5	10.3 ± 1.5
Follow-up	11.4 ± 1.8	10.9 ± 1.3	11.0 ± 1.4	10.9 ± 1.2
*p*	0.061	**<0.001**	**<0.001**	**<0.001**
ET (mm)	**Mean ± SD**	**Mean ± SD**	**Mean ± SD**	**Mean ± SD**
Pre-op	12.6 ± 5.5	13.7 ± 5.6	13.6 ± 5.5	13.5 ± 5.5
Post-op	6.3 ± 2.4	7.2 ± 2.6	6.3 ± 2.4	7.5 ± 3.3
*p*	**<0.001**	**<0.001**	**<0.001**	**<0.001**

Assessed using the Wilcoxon Signed-Rank Test.

Bold values indicate statistically significant within-group change from baseline to follow-up (*p* < 0.05; Wilcoxon signed-rank test).

In the <100 group, hemoglobin increased modestly (11.02 → 11.40 g/dL, *p* = .061), without reaching statistical significance. In contrast, patients with PBAC ≥ 100 showed a significant rise in Hb (10.10 → 10.92 g/dL, *p* < .001), suggesting greater hematologic benefit in those with heavier baseline bleeding.

ET decreased significantly in both groups: from 12.55 mm to 6.29 mm in the <100 group, and from 13.72 mm to 7.22 mm in the ≥100 group (both *p* < .001).

Among patients with follow-up PBAC < 100, hemoglobin increased significantly from 10.17 to 10.98 g/dL (*p* < 0.001). A similar rise was seen in the PBAC ≥ 100 group (10.22–10.93 g/dL; *p* < 0.001), indicating reduced blood loss in both groups, regardless of final bleeding status. Endometrial thickness also declined significantly post-treatment. In the PBAC < 100 group, values dropped from 13.64 to 7.04 mm (*p* < 0.001), while in the PBAC ≥ 100 group, they declined from 13.54 to 7.49 mm (*p* < 0.001).

**Figure 4:**
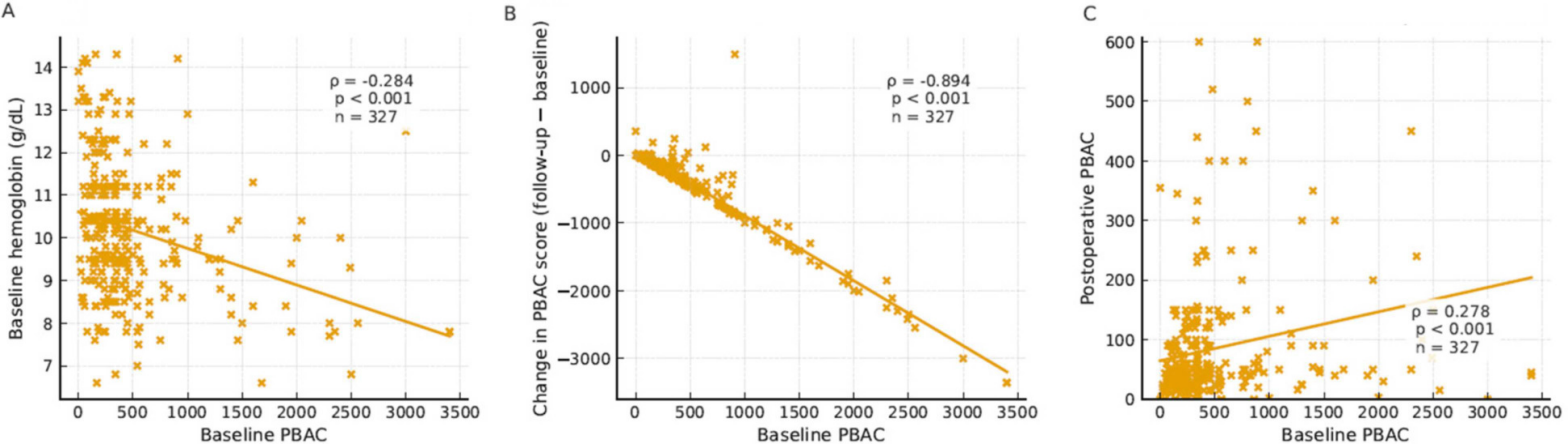
Scatter plots showing correlations between baseline PBAC and related parameters. **(A)** Baseline PBAC vs. baseline hemoglobin. **(B)** Baseline PBAC vs. change in PBAC score (follow-up−baseline). **(C)** Baseline PBAC vs. postoperative PBAC. Each dot represents one patient; solid lines show least-squares trend lines for visualization. Boxes report Spearman's *ρ*, *p*-value and *n* taken from Table 6 (*n* = 327 for all panels).

Patient-reported outcomes and complications by PBAC grouping are shown in Figure 1. When stratified by initial PBAC, the <100 group had a higher complication rate and lower proportions reporting satisfaction and willingness to recommend (*p* = 0.029, *p* = 0.016, and *p* = 0.013, respectively; Figure 1A; Table 3). In contrast, when stratified by postoperative PBAC, the proportions for complications, satisfaction, and recommendation did not differ significantly between groups (all *p* > 0.05; Figure 1B; Supplementary Figure S2). In Supplementary Figure S2, the 95% CIs for complications, satisfaction, and recommendation all cross 1.0, consistent with non-significant group differences. Exact percentages are printed on the bars. These groupwise findings complement the correlation analyses (Figure 4; Table 6), which indicate that higher baseline bleeding relates to greater improvement but only weakly to residual postoperative bleeding.

**Table 6:** Correlation between initial and follow-up PBAC scores and continuous variables.

Continuous variable		Initial PBAC Score	Follow-up PBAC Score
Initial PBAC Score	r	1	.278**
	P	.	**<0**.**001**
	n	327	327
Baseline Hb (g/dL)	r	−.284**	0.024
	p	**<0** **.** **001**	0.666
	n	**327**	327
Follow-up Hb (g/dL)	r	−.233**	−0.045
	p	<0.001	0.42
	n	327	327
Change in Hb (g/dL)	r	.157**	−0.077
	p	**0** **.** **004**	0.166
	n	**327**	327
Change in PBAC Score	r	−.894**	0.023
	p	**<0** **.** **001**	0.683
	n	**327**	327

Spearman's Rank Correlation Test.

Bold r and p values indicate statistically significant correlations (*p* < 0.05; Spearman's rank correlation).

** Indicates correlations that are statistically significant at the 0.01 level (*p* < 0.01; Spearman's rank correlation).

Baseline PBAC showed a moderate inverse correlation with baseline hemoglobin (*ρ* = −0.284, *p* < 0.001), a very strong negative correlation with change in PBAC score defined as follow-up−baseline (*ρ* = −0.894, *p* < 0.001), and a weak positive correlation with follow-up PBAC (*ρ* = 0.278, *p* < 0.001) (Figure 4; Table 6). Figure 4 visualizes these relationships with scatter plots and trend lines, while Table 6 lists the corresponding Spearman *ρ* estimates (*n* = 327 for each pair). Baseline PBAC also correlated negatively with follow-up hemoglobin (Table 6).

No significant correlations were detected between follow-up PBAC and other continuous variables (*p* > 0.05) in this dataset, suggesting that post-treatment bleeding levels were not closely associated with the examined clinical measures.

During follow-up, a total of 79 patients (24.2%) required reoperation. The main reasons were persistent abnormal uterine bleeding (*n* = 34, 10.4%), recurrence of intrauterine pathology such as polyp or myoma (*n* = 39, 11.9%), incomplete resection (*n* = 5, 1.5%), and other causes (*n* = 1, 0.3%).

Descriptive outcomes according to hysteroscopic procedure type, including PBAC score reduction, hemoglobin and endometrial thickness changes, complication distribution, and patient satisfaction are summarized in Table 7. Logistic regression analysis was performed to identify independent predictors of treatment response (defined as ≥50% reduction in PBAC score). As shown in Table 8, baseline PBAC score was a significant independent predictor of treatment response (*p* = 0.003). In contrast, age and BMI were not significantly associated with treatment outcome. Procedure type (polypectomy or myomectomy vs. endometrial resection) did not reach statistical significance. The wide confidence intervals observed in the combined procedure groups reflect the limited sample size in these subgroups.

**Table 7:** Descriptive outcomes by type of hysteroscopic procedure, including PBAC score reduction, hemoglobin and endometrial thickness changes, complication distribution, and patient satisfaction.

Procedure type	*n*	ΔPBAC (mean)	ΔHb (mean, g/dL)	ΔET (mean, mm)	Complication type (%)	Satisfaction (%)	
						Satisfied (*n*, %)	Not satisfied (*n*, %)
EMR	226	392.6	+0.74	6.8	None 95.1—Endometritis 0.9—Rupture 1.8—Bleeding 2.2	204 (90.3%)	22 (9.7%)
Polypectomy	53	358.6	+1.02	5.7	None 86.8—Endometritis 5.7—Rupture 1.9—Bleeding 5.7	41 (77.4%)	12 (22.6%)
Myomectomy	39	450.0	+0.95	5.7	None 94.9—Endometritis 0—Rupture 2.6—Bleeding 2.6	33 (84.6%)	6 (15.4%)
EMR+ Polypectomy	9	305.1	+0.07	5.1	None 88.9—Bleeding 11.1	7 (77.8%)	2 (22.2%)
EMR+ Myomectomy	2	145.0	−0.15	14.0	None 100	2 (100%)	0 (0%)

**Table 8:** Multivariable logistic regression analysis of predictors of treatment response (≥50% PBAC reduction).

Variable	OR	95% CI	*p*-value
Age	1.031	0.989–1.075	0.147
BMI	1.009	0.937–1.087	0.804
Baseline PBAC	**1** **.** **002**	**1.001** **–** **1.004**	**0** **.** **003**
Procedure: Polypectomy vs. EMR	0.839	0.353–1.993	0.691
Procedure: Myomectomy vs. EMR	0.508	0.214–1.206	0.125
Combined (EMR+ Polypectomy) vs. EMR	0.627	0.119–3.308	0.582
Combined (EMR+ Myomectomy) vs. EMR	4.7 × 10¹¹	0.000–∞	1.000

Bold values indicate statistically significant predictors of treatment response in the multivariable logistic regression model (*p* < 0.05).

## Discussion

The present study aimed to evaluate the clinical effectiveness of hysteroscopic interventions in abnormal uterine bleeding by analyzing changes in postoperative PBAC scores. In doing so, we also sought to investigate the usefulness of the PBAC scoring system as a practical and objective method for assessing treatment response based on improvements in menstrual bleeding, hemoglobin levels, and endometrial thickness.

The pre-to-postoperative reduction in PBAC and endometrial thickness together with the rise in hemoglobin indicates a consistent hematologic and anatomic response to treatment. These changes were large in magnitude and statistically robust across all three parameters (Figure 2; *p* < 0.001 for each paired comparison). This visualization complements the numerical summary by clarifying the direction and size of effect at a glance.

The distribution of procedures and histopathology (Figure 3) is consistent with routine AUB practice, with hysteroscopic treatment most often addressing benign intracavitary lesions. This case-mix supports the generalizability of our findings to similar gynecologic settings and helps explain the parallel improvements observed in PBAC, hemoglobin, and endometrial thickness.

Correlation findings (Figure 4). The correlation pattern supports the construct validity of the PBAC score. Higher baseline PBAC was associated with lower baseline hemoglobin (moderate inverse correlation), consistent with bleeding-related anemia, and with a larger absolute decrease in PBAC after hysteroscopy (very strong negative correlation with change defined as follow-up−baseline), indicating greater headroom for improvement in those with more severe preoperative bleeding. By contrast, the positive association between baseline and follow-up PBAC was weak in magnitude, suggesting that residual post-treatment bleeding is not determined solely by initial severity and may reflect procedural factors, underlying pathology, or patient-level variability. These relationships remained directionally consistent in sensitivity analyses and align with the expected clinical course following hysteroscopic treatment. Collectively, these correlations indicate that while baseline bleeding burden predicts anemia and the potential for improvement, it only weakly relates to residual bleeding, underscoring the role of procedural and pathologic determinants in post-treatment outcomes.

Our results exhibited a statistically significant decrease in PBAC scores following hysteroscopic intervention, with 77.2% of patients who were initially considered to have heavy menstrual bleeding (PBAC ≥ 100) dropping below this cut-off postoperatively. This confirms the efficacy of hysteroscopy for abnormal uterine bleeding through the elimination of intrauterine pathologies like polyps and submucous myomas (9, 10). Bleeding improvement was also paralleled by a significant hemoglobin increase, which was most pronounced in patients with higher initial PBAC scores, where mean hemoglobin increased from 10.1 to 10.9 g/dL. A positive correlation between PBAC decrease and hemoglobin increase (*ρ* = 0.157, *p* = 0.004), and a very strong inverse correlation between initial PBAC and PBAC change (*ρ* = −0.894, *p* < 0.001), demonstrated that individuals with the heaviest bleeding derived the most benefit. In contrast, the PBAC < 100 group only had a slight and statistically non-significant increase in hemoglobin. Moreover, increasing initial PBAC scores were moderately linked with lower pre- and postoperative hemoglobin values, illustrating the association between bleeding severity and anemia. These findings not only highlight the physiological advantages of hysteroscopic intervention, but also the usefulness of PBAC in objectively quantifying response to treatment (11).

A significant reduction in menstrual blood loss following hysteroscopic surgery was one of the most prominent observations of this study. The median PBAC score decreased significantly from 340 to 45 in the group with PBAC ≥ 100, and nearly 80% of the patients moved into the PBAC < 100 group after surgery. This corroborates existing evidence that removal of endometrial polyps or submucous fibroids through hysteroscopy significantly decreases menstrual quantity and improves quality of life. For example, van Dongen et al. noted a decrease in median PBAC score from 288 to 155 at six months after hysteroscopic polypectomy in premenopausal women with abnormal uterine bleeding (12). Likewise, Acharya et al. noted a dramatic fall in PBAC to about 65 one year after hysteroscopic treatment, with more than 80% of the patients recording complete or partial resolution of symptoms (*p* < 0.001) (13). A systematic review by Magnay et al. provides additional support for the clinical usefulness of PBAC as a valid and objective outcome measure for assessing treatment effect in patients with heavy menstrual bleeding (5).

Another important clinical finding in our study was the marked reduction in ET following hysteroscopic surgery. In both PBAC < 100 and PBAC ≥ 100 groups, median ET values decreased significantly post-surgery, in keeping with effective removal of intracavitary disease. Although there is limited literature reporting postoperative ET change in a longitudinal fashion, it is understood that removal of lesions via hysteroscopy is a factor in normalization of endometrial anatomy and symptom relief in AUB patients. In our cohort, the concordance between ET reduction, decreased PBAC scores, and hemoglobin level improvement provides validity for ET as a surrogate marker of procedural effectiveness in this context.

In addition to objective clinical endpoints, patient-reported outcomes in this study also serve to further highlight the therapeutic value of hysteroscopy. Most patients (87.5%) were satisfied with the procedure and 88.1% would undergo it again and recommend it to others. These observations highlight the real-world acceptability and perceived value of hysteroscopy from the patient's viewpoint. During follow-up, 24.2% of patients required re-intervention, most commonly hysterectomy or repeat treatment for persistent or recurrent AUB. This low retreatment rate provides support for the longevity of symptom control with hysteroscopy and is consistent with previous reports of high patient satisfaction and long-term efficacy with hysteroscopic polypectomy or myomectomy. Collectively, these findings affirm that hysteroscopy is both a clinically and experientially effective modality for the treatment of abnormal uterine bleeding.

Our cohort demonstrated a favorable safety profile. The overall postoperative complication rate was low (6.4%), most commonly postoperative bleeding (3.1%), with smaller proportions of uterine perforation and endometritis. The divergence between initial and postoperative PBAC groupings (Figures 1A,B) suggests that while baseline bleeding burden relates to complications and patient experience, post-treatment outcomes become broadly comparable across patients once the procedure is completed successfully (see also Table 3), indicating that patient experience and safety were broadly comparable regardless of residual bleeding. Although we did not detect between-group differences, case mix and procedural factors could plausibly influence complication profiles and merit evaluation in larger cohorts. This pattern was mirrored by effect-size estimates, with unadjusted ORs and 95% CIs overlapping unity for complications, satisfaction, and recommendation (Supplementary Figure S2), reinforcing the absence of clinically meaningful differences between postoperative PBAC groups. However, overall incidence of adverse events remained well within the acceptable bounds reported in the literature, where hysteroscopic procedures are typically associated with complication rates of less than 5%–7% in experienced hands (14, 15), supporting hysteroscopy as an effective and safe option for AUB when performed in appropriately selected patients.

In our subgroup analysis, BMI and baseline Hb levels were the only demographic variables with statistically significant group difference between the PBAC < 100 and PBAC ≥ 100 groups. Those with higher PBAC scores had significantly lower BMI and lower initial hemoglobin levels. These findings are perhaps reflective of the physiological consequences of chronic or excessive menstrual bleeding, namely iron-deficiency anemia and nutritional depletion, more likely to affect those with fewer body reserves. Higher BMI, however, has been associated in some studies with heaviness of menstrual bleeding due to estrogen excess and chronic endometrial stimulation, although the association is multifactorial and complex (16, 17). In our population, however, the reverse trend was observed. This would suggest that bleeding severity is more closely linked to local uterine pathology than the systemic hormonal milieu. These demographic associations underscore the need for individualized assessment in AUB, with consideration of both systemic and intrauterine factors implicated in bleeding burden.

There are a number of important strengths in this study. First, the sample size of 327 patients is quite large, allowing subgroup analysis with adequate statistical power. Second, both objective (PBAC score, hemoglobin, ET) and patient-reported outcomes (satisfaction, willingness to recommend) were measured, providing a comprehensive view of the clinical impact of hysteroscopic treatment. Last, the use of a standardized and validated bleeding assessment tool (PBAC) enhances the reproducibility and comparability of our findings. Furthermore, the fact that histopathological findings were included in the analysis provides the opportunity for correlation between severity of bleeding and particular intrauterine pathologies, providing richness to the interpretation of clinical outcomes. Some limitations should be acknowledged, however.

In subgroup comparisons, baseline PBAC emerged as the strongest indicator of symptomatic burden and potential for improvement, consistent with the clinical relevance of initial bleeding severity.

A *post-hoc* power analysis based on the observed effect size of PBAC reduction indicated that the achieved statistical power exceeded 0.90 with the sample of 327 patients, confirming adequacy of the sample size.

In total, our findings endorse the value of hysteroscopy in abnormal uterine bleeding management, consistent with study objectives. Although limited by the retrospective study design, the internal consistency of results across subgroups and the degree of change in PBAC scores and hemoglobin levels are indicative of a significant clinical benefit. The results are consistent with other research showing comparable improvement in bleeding and hematologic indices following hysteroscopic treatment. This enhances the external validity of our findings and underscores the value of PBAC as a useful outcome measure in everyday clinical practice.

The retrospective design clearly restricts the potential for determining causal relationships and might be subject to selection bias. Furthermore, although PBAC is an extremely commonly used measure, it is a semi-quantitative assessment and might be subject to reporting variation. Follow-up was restricted to short- to mid-term results; outcomes for longer-term recurrence and re-intervention rates were not obtainable. Data on hormonal status were also not obtainable, which would have offered additional insight into mechanisms of bleeding. Notwithstanding these restrictions, the study offers important evidence in favor of the clinical and patient-centered advantages of hysteroscopy in AUB management. Although the study was conducted in a single center, the findings may be generalizable to similar gynecological settings where hysteroscopic treatment of AUB is routinely performed.

## Conclusion

In summary, this research indicates that hysteroscopic therapy may help reduce menstrual blood loss and improve clinical outcomes in patients with abnormal uterine bleeding. Treatment-related changes in PBAC scores were associated with improvements in hemoglobin levels, endometrial thickness, and patient satisfaction. Importantly, baseline PBAC score emerged as the only independent predictor of treatment response, underscoring the clinical relevance of initial bleeding severity. These findings support the potential utility of PBAC as a simple and reliable tool for monitoring treatment response. Prospective, randomized controlled trials are warranted to validate these results and assess long-term outcomes.

## Data Availability

The raw data supporting the conclusions of this article will be made available by the authors, without undue reservation.

## References

[1] Wouk N Helton M. Abnormal uterine bleeding in premenopausal women. Am Fam Physician. (2019) 99(7):435–43. 30932448

[2] Vitale SG Watrowski R Barra F D’Alterio MN Carugno J Sathyapalan T Abnormal uterine bleeding in perimenopausal women: the role of hysteroscopy and its impact on quality of life and sexuality. Diagnostics. (2022) 12(5):1176. 10.3390/diagnostics12051176 35626331 PMC9140476

[3] Wortman M Daggett A Ball C. Operative hysteroscopy in an office-based surgical setting: review of patient safety and satisfaction in 414 cases. J Minim Invasive Gynecol. (2013) 20(1):56–63. 10.1016/j.jmig.2012.08.778 23107759

[4] Warner P Critchley HO Lumsden MA Campbell-Brown M Douglas A Murray G. Referral for menstrual problems: cross sectional survey of symptoms, reasons for referral, and management. Br Med J. (2001) 323(7303):24–8. 10.1136/bmj.323.7303.24 11440940 PMC34329

[5] Magnay JL O’Brien S Gerlinger C Seitz C. Pictorial methods to assess heavy menstrual bleeding in research and clinical practice: a systematic literature review. BMC Women’s Health. (2020) 20:1–15. 10.1186/s12905-020-0887-y 32041594 PMC7011238

[6] Higham JM O'brien P Shaw R. Assessment of menstrual blood loss using a pictorial chart. BJOG: Int J Obstet Gynaecol. (1990) 97(8):734–9. 10.1111/j.1471-0528.1990.tb16249.x 2400752

[7] Ko JK Lao TT Cheung VY. Pictorial Blood Loss Assessment Chart for Evaluating Heavy Menstrual Bleeding in Asian Women. 香港醫學雜誌. (2021). 10.12809/hkmj208743 34949729

[8] Janssen CA Scholten PC Heintz APM. A simple visual assessment technique to discriminate between menorrhagia and normal menstrual blood loss. Obstet Gynecol. (1995) 85(6):977–82. 10.1016/0029-7844(95)00062-V 7770270

[9] Deutsch A Sasaki KJ Cholkeri-Singh A. Resectoscopic surgery for polyps and myomas: a review of the literature. J Minim Invasive Gynecol. (2017) 24(7):1104–10. 10.1016/j.jmig.2017.08.645 28843536

[10] Giampaolino P Della Corte L Di Filippo C Mercorio A Vitale SG Bifulco G. Office hysteroscopy in the management of women with postmenopausal bleeding. Climacteric. (2020) 23(4):369–75. 10.1080/13697137.2020.1754389 32368939

[11] de Souza SS Camargos AF Ferreira MCF de Assis Nunes Pereira F de Rezende CP Araújo CAA Hemoglobin levels predict quality of life in women with heavy menstrual bleeding. Arch Gynecol Obstet. (2010) 281(5):895–900. 10.1007/s00404-009-1207-9 19693523

[12] Van Dongen H Janssen C Smeets M Emanuel M Jansen F. The clinical relevance of hysteroscopic polypectomy in premenopausal women with abnormal uterine bleeding. BJOG: Int J Obstet Gynaecol. (2009) 116(10):1387–90. 10.1111/j.1471-0528.2009.02145.x 19691630

[13] Acharya N Mishra P Mohammad S Karnik M Muneeba S Gemnani R Hysteroscopy as a therapeutic tool: a vision to spare the uterus in premenopausal abnormal uterine bleeding (AUB)/heavy menstrual bleeding (HMB), an update. Cureus. (2023) 15(10):e47877. 10.7759/cureus.47877 38021492 PMC10681274

[14] Jansen FW Vredevoogd CB Van Ulzen K Hermans J Trimbos JB Trimbos-Kemper TC. Complications of hysteroscopy: a prospective, multicenter study. Obstet Gynecol. (2000) 96(2):266–70. 10.1016/S0029-7844(00)00865-6 10908775

[15] Wright KN Hamilton K Kosturakis A. An overview of office hysteroscopy. Curr Obstet Gynecol Rep. (2024) 13(2):88–96. 10.1007/s13669-024-00377-y

[16] Mena GP Mielke GI Brown WJ. Prospective associations between physical activity and BMI with irregular periods and heavy menstrual bleeding in a large cohort of Australian women. Hum Reprod. (2021) 36(6):1481–91. 10.1093/humrep/deab055 33846724

[17] Fielder S Nickkho-Amiry M Seif MW. Obesity and menstrual disorders. Best Pract Res Clin Obstet Gynaecol. (2023) 89:102343. 10.1016/j.bpobgyn.2023.102343 37279629

