# Supplementary material for: Clinical utility of the PBAC score in quantifying treatment response to hysteroscopy: a retrospective observational study
Source: Front Surg. 2025 Nov 28;12:1684443. doi: 10.3389/fsurg.2025.1684443 (PMC12698563; doi:10.3389/fsurg.2025.1684443)

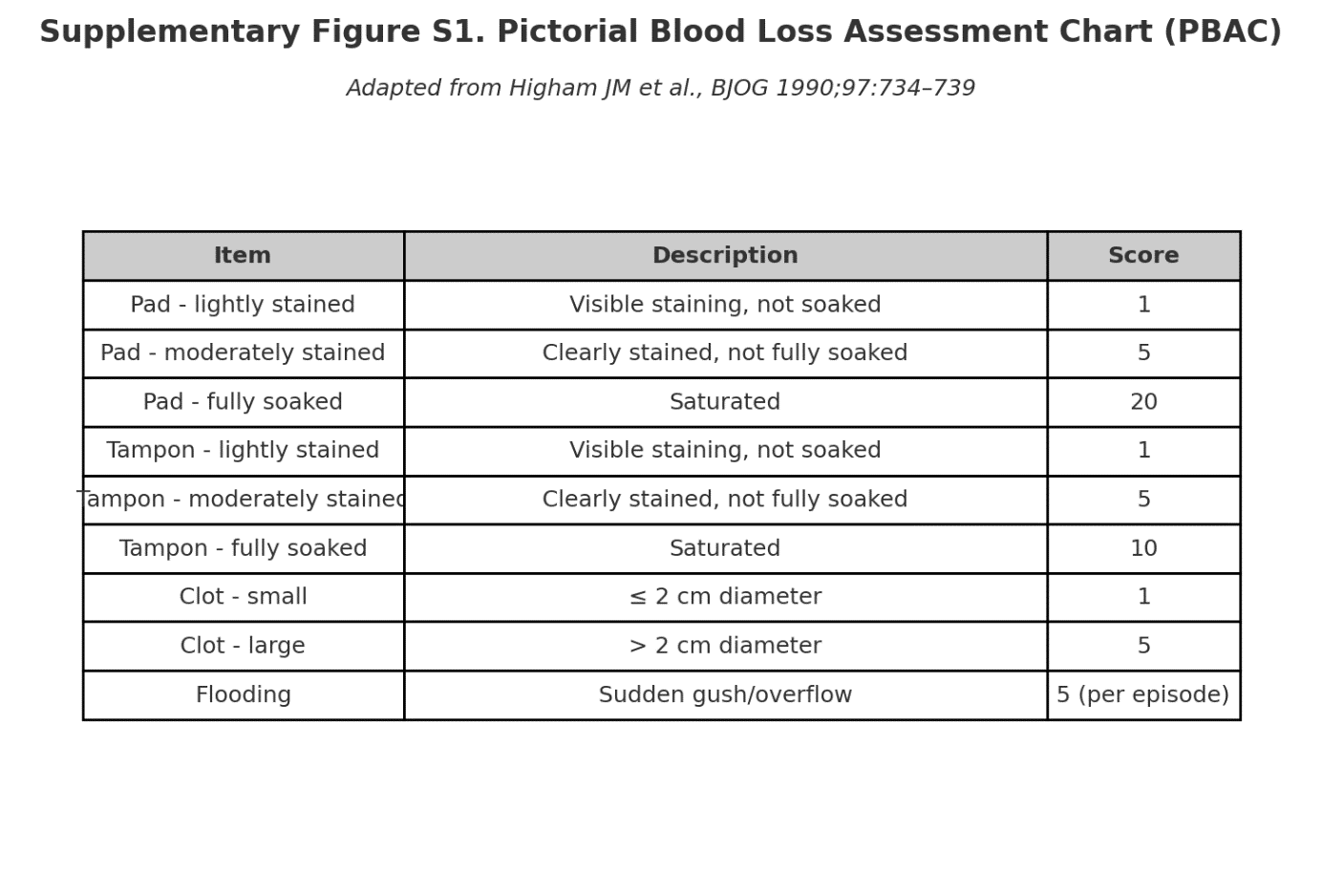


**Supplementary Figure S1.** Pictorial Blood Loss Assessment Chart (PBAC). This standardized scoring chart was used for patient self-assessment of menstrual blood loss. Scores are assigned according to the degree of saturation of sanitary products, presence and size of clots, and episodes of flooding. Higher PBAC scores indicate greater menstrual blood loss.


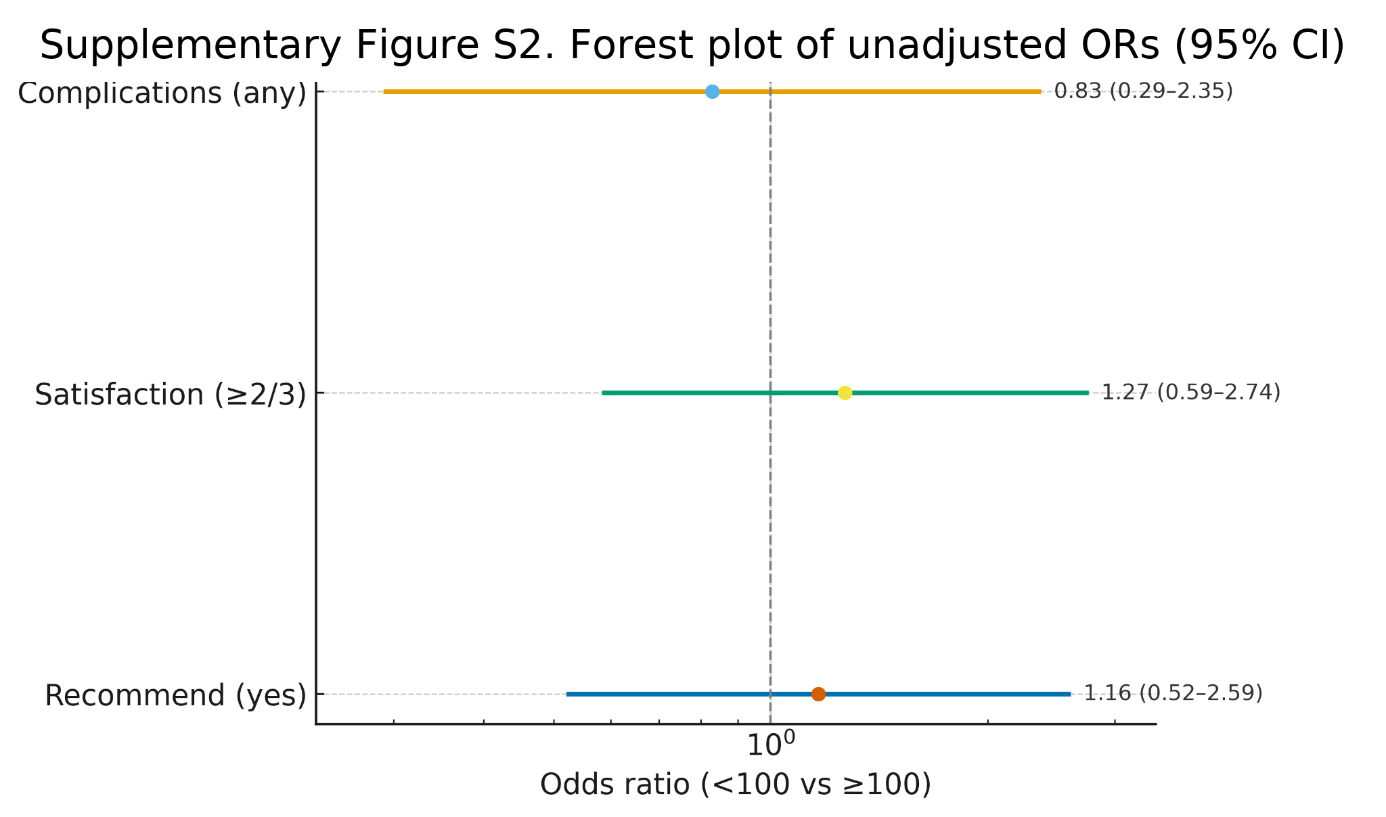


**Supplementary Figure S2.** Forest plot of unadjusted odds ratios (95% CI) comparing outcomes between postoperative PBAC groups (<100 vs ≥100). Outcomes were coded as Complications (any postoperative complication), Satisfaction (Likert 0–3; satisfied = 2–3), and Recommend (would undergo/recommend again = yes). Points show the OR for PBAC <100 relative to ≥100; horizontal bars indicate 95% CIs. The vertical dashed line marks OR = 1.0 (no difference). All CIs include 1.0, indicating no statistically significant between-group differences.


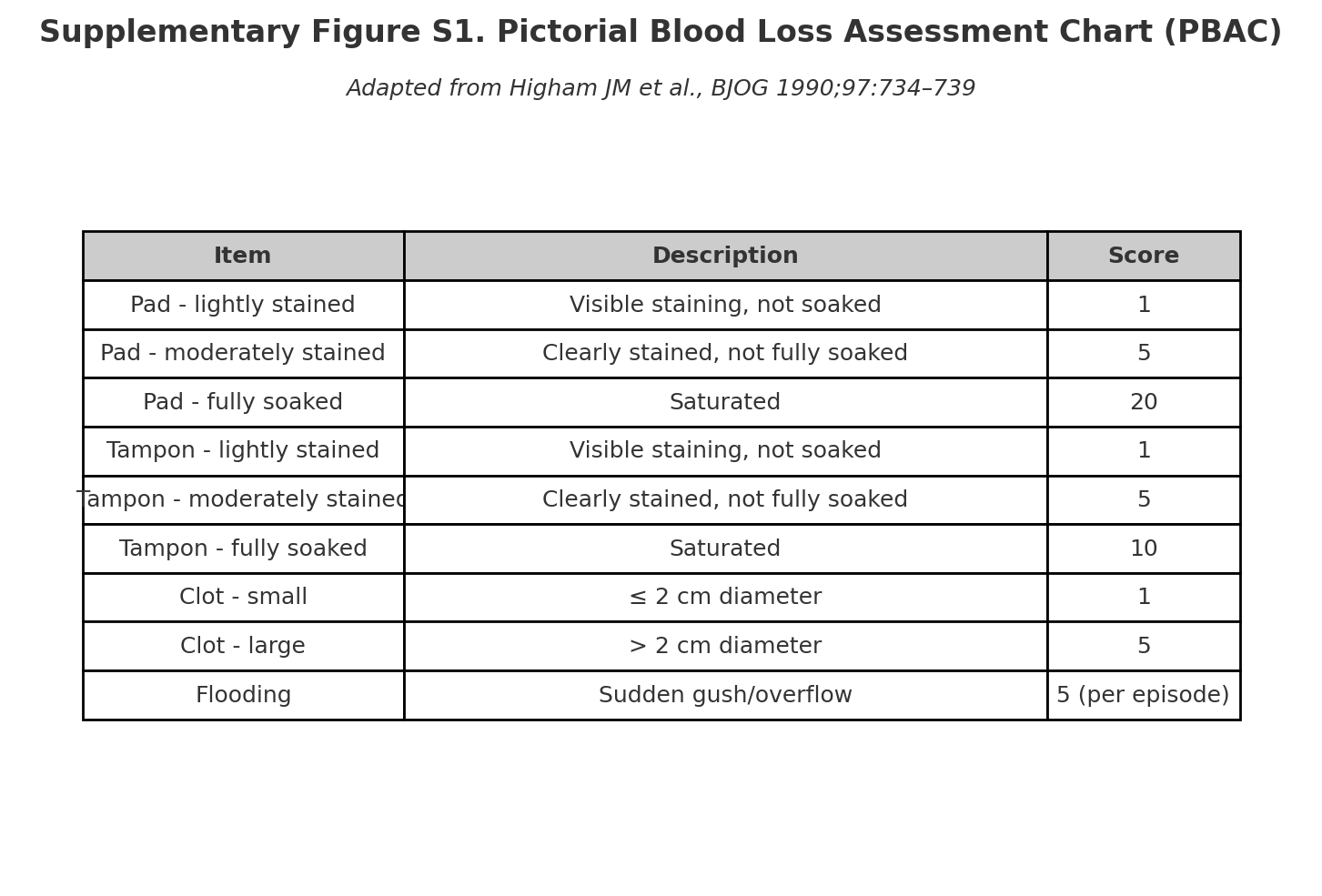

Supplement: Supplementary file 1 [file Supplementaryfile1.docx]
